# Supplementary material for: Content-rich biological network constructed by mining PubMed abstracts
Source: BMC Bioinformatics. 2004 Oct 8;5:147. doi: 10.1186/1471-2105-5-147 (PMC528731; doi:10.1186/1471-2105-5-147)
Supplement: Additional File 2 — The original results of the above study (non-essential files are deleted to keep the file size under the limit set by BMC bioinformatics). [file 1471-2105-5-147-S2.bz2 › chilibotAdditionalFile2/dip20/142ID7739537E502/html/RBL1_TFDP1.html]

 


 **RBL1** and **TFDP1** 
  
Found 64 abstracts in PubMed, retrieved 20.  
 

 What does Google say? 
 PDF only 
| .edu only 

---

**Interactive relationship** (e.g. stimulation, inhibition, etc)

**Neutral relationship**- During late G1 and S phase this complex is replaced by at least three different E2F complexes, one of which is an E2F complex containing p107  [ **RBL1** ]  or pRB  [ **RBL1** ]  as well as two free E2F complexes consisting of E2F4 DP1  [ **TFDP1** ]  and E2F1 3 DP1  [ **TFDP1** ] .  Ref: 10540350 Eur J Immunol, 1999

**Non-interactive relationship** (e.g. studied together, co-existance, homology, etc.)

- Western blot analyses of whole cell extracts revealed that amounts of E2F4, E2F1, DP1  [ **TFDP1** ] , and p107  [ **RBL1** ]  remained unchanged after infection of C33 cells.  Ref: 10366563 Virology, 1999
- Antibodies to DP 1  [ **TFDP1** ] , E2F1 to 5, p107  [ **RBL1** ] , or pRb  [ **RBL1** ]  failed to either supershift or block E2Fmyb sp complex formation.  Ref: 10567569 Mol Cell Biol, 1999
- Because Cdk4 6 phosphorylates retinoblastoma protein pRb  [ **RBL1** ]  family members that then modulate the transcriptional activity of E2F DP1  [ **TFDP1** ]  complexes, we examined the involvement of these components in DNA damage evoked neuronal death.  Ref: 10777774 J Neurosci, 2000
- Expression of pRb  [ **RBL1** ] , cyclin D1, cdk4, cyclin E, cdk2, E2F1 and DP 1  [ **TFDP1** ]  was determined in MCF 7 and MDA MB 468 breast carcinoma cells, H460 and Calu 6 non small cell lung carcinoma cells, H82 and SW2 small cell lung carcinoma cells, HCT116 and HT29 colon carcinoma cells and LNCaP and DU 145 prostate carcinoma cells.  Ref: 11052627 Cancer Chemother Pharmacol, 2000
- In serum stimulated S phase cells, the composition of the complex switched to E2F1 4, DP1  [ **TFDP1** ]  and p107  [ **RBL1** ] , together with cyclin A and cyclin dependent kinase 2.  Ref: 12139485 Biochem J, 2002
- Expression of pRB  [ **RBL1** ] , cyclin cyclin dependent kinases and E2F1 DP 1  [ **TFDP1** ]  in human tumor lines in cell culture and in xenograft tissues and response to cell cycle agents.  Ref: 11052627 Cancer Chemother Pharmacol, 2000
- Release of E2F1 DP1  [ **TFDP1** ]  heterodimers from repression mediated by the retinoblastoma tumor suppressor pRB  [ **RBL1** ]  triggers cell cycle entry into S phase, suggesting that E2F1 and DP1  [ **TFDP1** ]  proteins must act in unison, either to facilitate or to suppress cell cycle progression.  Ref: 12588846 Development, 2003
- Overexpression of c myc, Cyclins D1, E, and A, and E2F1 genes, at messenger RNA mRNA and protein levels, rise in Cyclin D1 CDK4, Cyclin E CDK2, and E2F1 DP1  [ **TFDP1** ]  complexes, and pRb  [ **RBL1** ]  hyperphosphorylation occurred in nodules and HCCs of F344 rats.  Ref: 12029619 Hepatology, 2002
- A complex containing Smad3, the transcription factors E2F4 5 and DP1  [ **TFDP1** ] , and the corepressor p107  [ **RBL1** ]  preexists in the cytoplasm.  Ref: 12150994 Cell, 2002
- Furthermore, E2F species were sequestered in complexes with p107  [ **RBL1** ] , p130, DP 1  [ **TFDP1** ] , and cyclins A and E.  Ref: 12234612 Biochem Pharmacol, 2002
- Moreover, introduction of constitutively active pRb  [ **RBL1** ]  and transcriptionally inert mutant E2F1 DP1  [ **TFDP1** ]  efficiently protected cardiomyocytes from apoptosis.  Ref: 12411392 Circ Res, 2002
